# Value-Driven Adaptations of Mesolimbic Dopamine Release Are Governed by Both Model-Based and Model-Free Mechanisms

**DOI:** 10.1523/ENEURO.0223-24.2024

**Published:** 2024-07-03

**Authors:** Rhiannon Robke, Tara Arbab, Rachel Smith, Ingo Willuhn

**Affiliations:** ^1^The Netherlands Institute for Neuroscience, Royal Netherlands Academy of Arts and Sciences, Amsterdam 1105BA, The Netherlands; ^2^Department of Psychiatry, Amsterdam University Medical Centers, University of Amsterdam, Amsterdam 1105AZ, The Netherlands

**Keywords:** behavior, dopamine, learning, motivation, nucleus accumbens, rats

## Abstract

The magnitude of dopamine signals elicited by rewarding events and their predictors is updated when reward value changes. It is actively debated how readily these dopamine signals adapt and whether adaptation aligns with model-free or model-based reinforcement–learning principles. To investigate this, we trained male rats in a pavlovian-conditioning paradigm and measured dopamine release in the nucleus accumbens core in response to food reward (unconditioned stimulus) and reward-predictive conditioned stimuli (CS), both before and after reward devaluation, induced via either sensory-specific or nonspecific satiety. We demonstrate that (1) such devaluation reduces CS-induced dopamine release rapidly, without additional pairing of CS with devalued reward and irrespective of whether the devaluation was sensory-specific or nonspecific. In contrast, (2) reward devaluation did not decrease food reward-induced dopamine release. Surprisingly, (3) postdevaluation reconditioning, by additional pairing of CS with devalued reward, rapidly reinstated CS-induced dopamine signals to predevaluation levels. Taken together, we identify distinct, divergent adaptations in dopamine-signal magnitude when reward value is decreased: CS dopamine diminishes but reinstates fast, whereas reward dopamine is resistant to change. This implies that, respective to abovementioned findings, (1) CS dopamine may be governed by a model-based mechanism and (2) reward dopamine by a model-free one, where (3) the latter may contribute to swift reinstatement of the former. However, changes in CS dopamine were not selective for sensory specificity of reward devaluation, which is inconsistent with model-based processes. Thus, mesolimbic dopamine signaling incorporates both model-free and model-based mechanisms and is not exclusively governed by either.

## Significance Statement

Although it is well known that dopamine plays a principal role in reward learning, the temporal dynamics and the associated theoretical framework of the dopamine response to changing reward values are debated. Most studies conceptualize and classify dopamine signals as governed exclusively by either model-based or model-free processes. However, our work shows involvement of both processes: the temporal dynamics of dopamine response to conditioned stimuli appear model-based, and the persistence of reward-evoked dopamine to the reward itself appears model-free. The implication of our findings is that either model-free or model-based dynamics can operate in a mixed framework or that these reinforcement-learning concepts are not apt in describing the activity of the mesolimbic dopamine system in this experimental context.

## Introduction

When we encounter rewarding stimuli in our environment, we heed the circumstances that surround them, learning to associate rewards with their predicting stimuli. During this learning, the mesolimbic dopamine system develops pronounced responses to these outcome predictors (Schultz et al., 1997; Day et al., 2007) as a function of the subjective value of these rewarding events (as well as other factors): the greater the palatable value of the reward, the more pronounced the dopamine system responds to the reward itself and its conditioned cue that predicts it (Tobler et al., 2005; Syed et al., 2016; van Elzelingen et al., 2022a). Dopamine-system activity can be reliably evaluated as the amount of dopamine released from dopamine-neuron terminals in the striatum (Mohebi et al., 2019); the ventromedial mesolimbic striatum, specifically the nucleus accumbens, exhibits the largest release in response to food rewards (van Elzelingen et al., 2022a,b).

The subjective value of palatable rewards is influenced by conditions such as internal states (e.g., appetite) and external context (e.g., reward abundance). When such conditions change, the subjective value of reward is updated. The nature of this updating process depends on the brain's representation (or internal model) of the world. In this context, there is an ongoing debate about how the dopamine system adapts to changing conditions, where it has been suggested that adaptations follow either model-free (Sutton and Barto, 1987; Montague et al., 1996; Schultz et al., 1997) or model-based reinforcement learning (Sharpe et al., 2017, 2020) or a mixture of the two (Starkweather et al., 2017; Starkweather and Uchida, 2021). Model-free processes require no substantive representation of the world and instead are thought to retrospectively update so-called cached (or past) values after repeated experience of the altered value conditions; this process is low in computational cost but proceeds relatively slowly (Sutton and Barto, 1987; Doya, 1999; Dickinson and Balleine, 2002; Daw et al., 2005), typically via a so-called temporal-difference reward–prediction error (RPE) that does not incorporate stimulus identity (Sutton and Barto, 1987; Dayan and Berridge, 2014). In contrast, model-based processes require a more complete map-like model of the world that incorporates stimulus identity and can perform updates off-line, i.e., without experiencing changed value conditions (Sutton and Barto, 1987; Daw et al., 2005; de Wit and Dickinson, 2009; Dayan and Berridge, 2014); this process is computationally more costly as it involves prospective cognition, is more precise, and updates faster than model-free processes.

The subjective value of palatable (food) reward is readily manipulated by inducing sensory-specific satiety: devaluing the reward by providing access to excessive amounts of the specific food in question. This type of devaluation has mainly been used to determine whether instrumental behavior is habitual (Dickinson, 1987; Watson et al., 2022), a concept that bears similarities to model-free behavior.

There is evidence to indicate that sensory-specific satiety devaluation may reduce subsequent dopamine release off-line (without the requirement of reexperiencing the devalued reward; Aitken et al., 2016; Papageorgiou et al., 2016). This dopamine decrease is consistent with reports demonstrating that satiety diminishes both burst firing of dopamine neurons (Branch et al., 2013) and reward-evoked dopamine release in the nucleus accumbens core (Cone et al., 2014), as well as overall dopamine release measured on a slower time scale with microdialysis (Ahn and Phillips, 1999; Ostlund et al., 2011). Others have found that food-predictive cues elicit dopamine release independent of internal state (e.g., hunger or satiety; Gómez-A et al., 2022). Taken together, although there is clear evidence that satiety affects dopamine, the specifics are insufficiently understood.

To resolve the role of model-free and model-based processes in dopamine signaling, we trained male Long–Evans rats in a pavlovian-conditioning paradigm and measured dopamine release in the nucleus accumbens core with fast-scan cyclic voltammetry (FSCV) in response to food reward [unconditioned stimulus (US)] and reward-predictive conditioned stimuli (CS) before and after reward sensory-specific or nonspecific devaluation. Our findings indicate that value-based changes in mesolimbic dopamine signaling are consistent with aspects of both model-based and model-free mechanisms.

## Materials and Methods

### Animals

Adult male Long–Evans rats (200–300 g; Janvier Labs) were individually housed and kept on a reversed 12 h light/dark cycle (lights on 2100–0900) with controlled temperature and humidity. All animal procedures were in accordance with Dutch and European law and approved by the Animal Experimentation Committee of the Royal Netherlands Academy of Arts and Sciences.

In total, 35 rats underwent surgery (bilateral FSCV electrode implantation), 20 of which were included in the study with at least one functional FSCV electrode that was histologically verified in its anatomical target (nucleus accumbens core). Animals were excluded if FSCV electrodes were implanted outside the target area and due to methodological challenges such as electrical noise during FSCV recordings or accidental disconnection of the recording equipment. Data from only one electrode per animal were used in each analysis; however, if that electrode became compromised in the course of experiments, we used the other electrode, provided it was implanted in the nucleus accumbens core (four animals in total). All rats were food restricted to 85% of their *ad libitum*-feeding body weight, and water was provided *ad libitum*.

### Stereotaxic surgery

Thirty minutes prior to surgery, the analgesic Metacam (0.2 mg meloxicam/100 g) was injected subcutaneously. Rats were induced under isoflurane anesthesia, placed into the stereotaxic frame where body temperature was monitored and maintained on an isothermal pad, and the scalp was shaved and disinfected using 70% ethanol. Upon incision of the scalp, lidocaine was applied (100 mg/ml). Holes were drilled for four surgical screws, an Ag/AgCl reference electrode targeting the forebrain, and two custom-made carbon–fiber microelectrodes bilaterally targeting the nucleus accumbens core (1.2 mm AP, 1.5 mm ML, and −7.1 DV; Paxinos and Watson, 2007). Electrodes were secured and anchored to the surgical screws with dental acrylic cement. Following surgery, rats received a 2 ml subcutaneous saline injection and were placed in a temperature-controlled recovery chamber to be monitored. Rats recovered from surgery for 2 weeks before food restriction, behavioral training, and recording.

### Behavioral procedures

All behavioral experiments were conducted in modified operant boxes (32 × 30 × 29 cm, Med Associates), equipped with a food magazine (connected to an automated food-pellet dispenser), a house light, one retractable lever opposite the food magazine, multiple tone generators, and metal grid floors (Med Associates). Each operant box was surveilled by a video camera. The boxes were housed in metal Faraday cages that were insulated with sound-absorbing polyurethane foam.

Experimental pellets contain 21% protein, 13.8% fat, and 65.5% carbohydrates for a caloric value of 3.7 kcal/g; the homecage chow is made up of 24% protein, 18% fat, and 58% carbohydrates for a caloric value of 3.1 kcal/g.

#### Experiment 1: validation of the devaluation procedure

A cohort of rats (*n* = 8) was used to determine the influence of our feeding regime on motivation by measuring the rats’ lever-press rate in an operant task using a fixed-interval 5 (FI5) reinforcement schedule with discrete trials. Prior to the experiment, animals were trained to lever press for a pellet twice daily. The training steps and criteria to advance to the next training step during lever-press training were as follows: (1) fixed-ratio 1 (FR1) with <2 s latency to lever press as a criterion; (2) FR3 with <5 s latency to rewarding lever press as a criterion; (3) and lastly, FI5 lever access with <5 s latency to rewarding lever press as the final criterion. All animals completed their training within 1 week. On experiment days, animals were placed into the operant chamber, and within the same session, the rat's motivation was first tested in a hungry state, followed by satiety, separated by a 30 min feeding (devaluation) period. During this feeding period, animals received either (1) no food (mock), (2) 20 g of sucrose pellets (experimental diet), or (3) 20 g of chow (homecage diet) within the operant chamber. Before and after the 30 min feeding period, animals performed 15 trials with lever access on a FI5 schedule, separated by a variable intertrial interval averaging 30 s (vITI30; range, 15–45 s), or 15 total minutes, whichever came first. A lever press after the 5 s interval following lever insertion led to lever retraction and the immediate delivery of a food pellet. Lever presses prior to the end of this 5 s interval were without programmed consequences. We compared the lever-press rate between hungry and sated conditions. Analysis of operant behavior such as the number of head entries into the food magazine and lever presses were registered via an automated procedure (Med Associates) and analyzed in MATLAB R2022b (MathWorks).

#### Validation of the pavlovian-conditioning procedure

Rats (*n* = 20) were placed in the operant box and trained to distinguish between two distinct 5 s audio tones (CS+ and CS−; 3 and 8 kHz at 75 dB; counterbalanced between rats) for up to 120 trials daily (max. 60 min). The probability for each trial type (CS+ or CS−) was 50%, and they were presented in randomized order, separated by a vITI30 (range, 15–45 s). For CS+ presentations, a pellet was delivered immediately after the cue offset, whereas the CS− was never paired with any reward. The rats were trained until their magazine-entry rate stabilized for 3 consecutive days (∼10–15 d of training).

#### Experiment 2: effects of devaluation on dopamine response to CS and US

Following extensive training to distinguish CS+/− presentations, rats (*n* = 14 for Fig. 2, *n* = 20 for Fig. 3) were placed in the operant chamber and tethered to the FSCV recording setup to determine the effect of hunger state on nucleus accumbens core dopamine release in response to CS+ presentations. Within the same recording session, rats were tested in both hungry and sated states, as previously described (Experiment 1). In each state, animals experienced three consecutive CS+ only trials (in which the predictive 5 s audio tone did not result in reward delivery), followed by 50 conditioning trials in which the 5 s audio tone was followed by reward delivery (CS+; 70% of trials) or reward delivery never followed (CS−; 10% of trials) and probe trials, in which the CS+ was presented alone (10%) or an experimental food pellet was delivered alone (US; 10% of trials). In Figure 2, data from 14 animals were used, instead of 20 (Fig. 3), because one cohort of animals did not experience three consecutive “CS+ only” trials (in their hungry state). These trials were added only in subsequent cohorts. Probe trials were used to determine whether dopamine was related to reward outcomes or RPE. All conditioning trials were presented in pseudorandomized order and separated by a vITI30 (range, 15–45 s). All rats underwent exposure to mock [*n* = 13 (Fig. 2) or *n* = 17 (Fig. 3)], pellet [*n* = 11 (Fig. 2) or *n* = 17 (Fig. 3)], and chow [*n* = 12 (Fig. 2) or *n* = 16 (Fig. 3)] treatments, and the sequence of exposure to each feeding regimen was counterbalanced across animals. We compared the average dopamine concentration during CS+ presentation (0–5 s; after the cue onset) across feeding conditions. In probe trials (unpredicted pellets or omitted outcome), we compared dopamine transients during relevant epochs. The positive peak (max) dopamine concentration over 5 s following the event of interest (i.e., CS+ presentation, US delivery) was compared between hungry and sated conditions.

### FSCV measurements and analysis

As described previously (Willuhn et al., 2014), FSCV was used to detect subsecond changes in extracellular dopamine concentration using chronically implanted carbon–fiber microelectrodes. Prior to recording, microelectrodes were connected to a head-mounted voltammetric amplifier, interfaced with a PC-driven data acquisition and analysis system (National Instruments) through an electrical commutator (Crist), which was mounted above the test chamber. Every 100 ms, voltammetric scans were repeated to achieve a sampling rate of 10 Hz. A triangular waveform from −0.4 to +1.3 V back to −0.4 V at 400 V/s (vs the implanted Ag/AgCl reference electrode) was applied to the working electrode. Chemometric analysis with a standard training set was used to isolate dopamine from the voltammetric signal (Clark et al., 2010). All data were smoothed with a 10-point median filter, and baseline (set at 1 s before event of interest) subtraction was performed on a trial-by-trial basis prior to analysis of average concentration.

### Histological verification of recording and stimulation sites

After completion of the experiments, rats were deeply anesthetized using a lethal dose of pentobarbital. FSCV recording sites were marked in vivo by electrolytic lesion before transcardial perfusion with saline followed by 4% paraformaldehyde (PFA). Brains were removed and postfixed in PFA for 24 h after which they were placed in 30% sucrose for cryoprotection. The brains were rapidly frozen using an isopentane bath, sliced on a cryostat (40 μm coronal sections, −20°C), and stained with cresyl violet. All animals included in this study had electrodes positioned in the nucleus accumbens core.

### Statistical analysis

FSCV and behavioral data were analyzed using one- or two-tailed paired or unpaired *t* tests, repeated measures ANOVAs, or their nonparametric equivalents when appropriate. Statistical analysis was carried out using Prism (GraphPad Software) and MATLAB R2022b (MathWorks) with statistical significance set at *p* < 0.05. Individual electrochemical signals were averaged across trials within a session within animals and subsequently across animals. Graphical representations were made using Prism and MATLAB. All data are presented as mean + or mean ± SEM.

## Results

### Behavior during devaluation test and pavlovian conditioning

**Figure 1.**
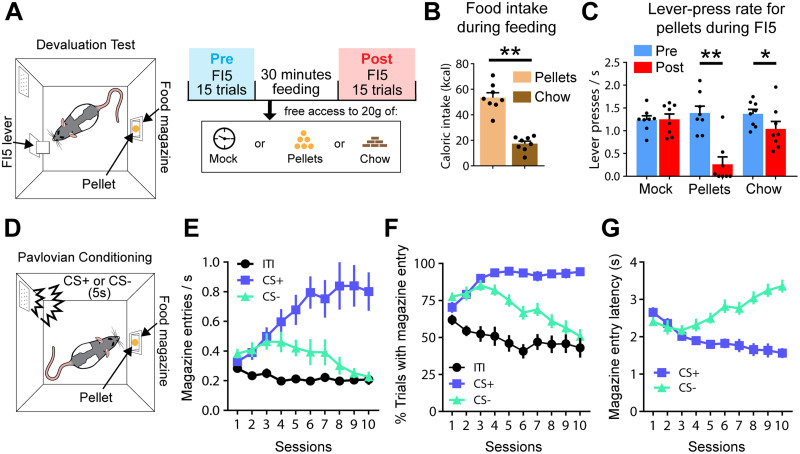
Behavior during devaluation test and pavlovian conditioning. ***A***, Left, Schematic of operant task in which a lever is extended for FI5, requiring a press after this period for the delivery of a food pellet in the food magazine. The task concluded after the completion of 15 trials or after 15 min, whichever occurred first. Right, Motivation to lever press for reward was tested before (blue) and after (red) a 30 min feeding period. On separate days, the rats received either no food (mock) or 20 g of pellets or chow within the operant chamber (exposure to each feeding regimen counterbalanced across animals). Chow is the homecage diet, whereas pellets are only presented in the operant chamber. ***B***, Caloric intake during pellet- or chow-feeding periods. ***C***, Lever presses during the FI5 period in hungry (blue) and sated (red) states across mock, pellet, and chow-feeding conditions. ***D***, In our pavlovian task, a 5 s audio-tone cue (3 and 5 kHz tones; counterbalanced) is presented on a vITI30 schedule, followed by either a single pellet (CS+) or no reward (CS−). ***E***, Food-magazine head–entry rate during the ITI (black), CS+ (blue), and CS− (green) presentations across conditioning sessions. ***F***, The percentage of trials with a food-magazine head–entry during the ITI (5 s prior to cue onset) and cue epochs. ***G***, Latency of food-magazine head–entry upon cue onset. *p < 0.05; **p < 0.01.

To assess motivation to work for reward, rats were trained to lever press for pellet delivery on an FI5 reinforcement schedule (Fig. 1*A*). All animals (*n* = 8) were tested in both a hungry and sated state within the same session, separated by a 30 min feeding period in which they received either no food (mock) or 20 g of pellets (experimental food) or chow (homecage diet) inside the operant chamber (Fig. 1*A*). During this feeding period, rats consumed large amounts of the offered food, with calorie intake on average being higher for pellets than chow (*W* = 36; *p* = 0.0078; Fig. 1*B*). However, both feeding treatments significantly affected behavior, reflected by a decrease in lever-press rate during the postfeeding FI5 period for both pellets (*W* = −26; *p* = 0.0078) and chow (*W* = −29; *p* = 0.0469), but not mock (*W* = 1; *p* = 0.9688; Fig. 1*C*). Thus, both our sensory-specific devaluation procedure and general satiety are sufficient to decrease motivation to lever press for pellet delivery.

**Figure 2.**
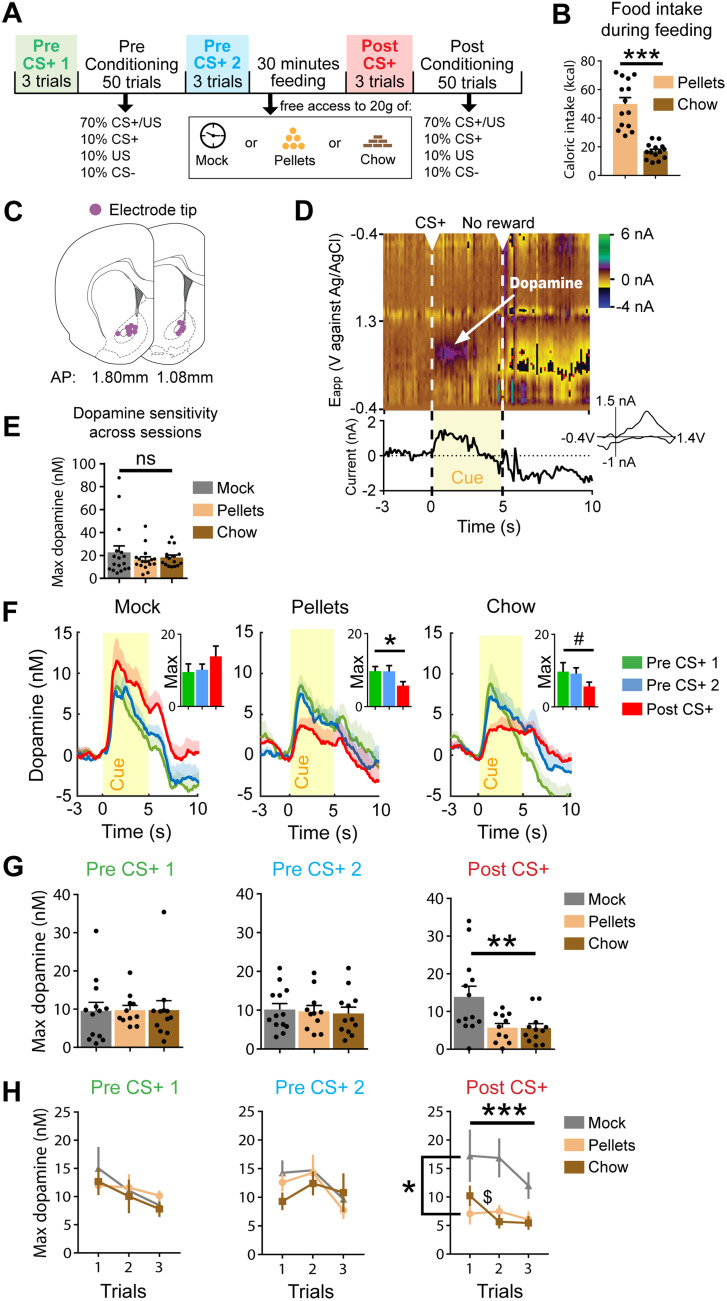
Dopamine response to CS+ is rapidly reduced after satiety. ***A***, Trial-based schematic where consecutive CS+ presentations (without reward pairing) are evaluated pre- (green, blue) and post- (red) feeding. ***B***, Caloric intake during pellet- or chow-feeding periods. ***C***, Histological verification of electrode position in the nucleus accumbens core (*n* = 24 electrodes, 20 animals). ***D***, Top, Representative color plot and dopamine response to CS+. Dotted lines denote the CS+ onset and offset in an omission trial where no reward was given. Bottom left, Current versus time trace shows dopamine release in response to the CS+ (yellow indicates duration of CS+ presentation). Bottom right, Cyclic voltammogram confirms the detection of dopamine. ***E***, Electrode sensitivity to dopamine, prompted by unexpected single-pellet deliveries before the start of recording sessions, remains stable across sessions. ***F***, Dopamine release for CS+ presentations in mock (left), pellets (middle), and chow (right) feeding conditions. Dopamine concentrations for pre- and postfeeding CS+ presentations are shown as insets. ***G***, Dopamine concentration compared across feeding conditions for pre-CS+ 1, pre-CS+ 2, and post-CS+ presentations. While consistent across treatments before devaluation, dopamine response to CS+ after devaluation significantly decreased after pellet and chow consumption compared with mock treatment. ***H***, Dopamine concentration per trial for pre-CS+ 1, pre-CS+ 2, and post-CS+ presentations across feeding treatments. *p < 0.05; **p < 0.01; ***p < 0.0001; ^#^*p* < 0.1; ^$^*p* < 0.05 (chow, T1 vs T2); ns, not significant.

Next, rats (*n* = 20) were trained (pavlovian conditioning) to distinguish between two 5 s audio tones: a CS+ that signaled reward delivery and CS− resulting in no reward delivery (Fig. 1*D*). Animals learned to reliably distinguish between CS+ and CS−, indicated by (1) divergently increased and decreased magazine entries in response to CS+ and CS−, respectively (Fig. 1*E*); (2) divergently increased and decreased percentage of CS+ and CS− trials resulting in magazine entry, respectively (Fig. 1*F*); and (3) divergently increased and decreased magazine-entry latency during CS+ and CS− presentations, respectively (Fig. 1*G*). As another crucial validation of task acquisition, by the end of the pavlovian training period, magazine entries elicited by CS− presentation no longer differed from baseline responding rates (during the ITI period; Fig. 1*E*,*F*).

### Dopamine response to CS+ is promptly reduced by devaluation

After pavlovian conditioning, we evaluated how satiety affects dopamine response to CS+ presentations (Fig. 2*A*). Rats (*n* = 14) were presented three unrewarded CS+ trials (pre-CS+ 1), followed by a conditioning phase (preconditioning) and then another three unrewarded CS+ presentations in both hunger (pre-CS+ 2) and sated states (post-CS+), separated by the aforementioned 30 min feeding period. During this feeding period, rats consumed large amounts of the offered food, with calorie intake on average being higher for pellets than chow (*W* = −105; *p* = 0.0001; Fig. 2*B*). Extracellular dopamine fluctuations were measured during pre-CS+ 1, pre-CS+ 2, and post-CS+ trials using FSCV, with chronic electrodes targeting the nucleus accumbens core (Fig. 2*C*). Dopamine was reliably released in response to CS+ presentation (representative color plot in Fig. 2*D*) as well as stably released across sessions in response to unpredicted pellet delivery (performed as a control for electrode integrity before the start of each session); there was no difference between groups (*H*_(49)_ = 0.5321; *p* = 0.7664; Fig. 2*E*).

To understand how value-based changes to a CS+ influence dopamine dynamics, we compared max (peak) dopamine release during the unrewarded 5 s CS+ presentations within feeding treatment groups. Irrespective of whether the devaluation was sensory-specific or nonspecific (Fig. 2*F*), we found a general decrease in dopamine release to the CS+ postfeeding compared with prefeeding for pellets (*x*^2^_(10)_ = 7.818; *p* = 0.0187) and a trending decrease for chow (*x*^2^_(11)_ = 4.667; *p* = 0.0970). We did not observe a significant correlation between post-CS+ max dopamine and caloric intake of chow (*r* = 0.2952; *p* = 0.2670; data not shown), suggesting the amount of calories consumed was not a crucial driving factor in reaching satiation or decreasing dopamine after devaluation. There was no difference for the mock group (*x*^2^_(12)_ = 2; *p* = 0.3679). Prior to the feeding treatment, there was no change in dopamine release for the CS+ trials between pre-CS+ one and pre-CS+ two devaluation treatments; however after access to unlimited food, we observed a significant decrease in post-CS+ averaged dopamine release for both pellet and chow-feeding treatments (main effect of group, *F*_(2,33)_ = 5.827; *p* = 0.0068; Fig. 2*G*). Post hoc analysis showed that both pellet and chow devaluation differed from mock (mock vs pellets, *p* = 0.0191; mock vs chow, *p* = 0.0145).

As both of our devaluation procedures (pellets and chow) decreased motivation to work for reward delivery (Fig. 1*C*) and rapidly (*n* = 3 trials) decreased dopamine release during post-CS+ trials (Fig. 2*G*), we hypothesized that unlimited access to food promptly updates dopamine dynamics in a model-based manner, in which case this value-based change would be induced by the first post-CS+ trial, without the need for additional exposure to the CS+. Indeed, feeding affected dopamine release across post-CS+ trials (Fig. 2*H*; main effect of group, *F*_(2,91)_ = 11.27; *p* < 0.0001; main effect of trial, *F*_(2,91)_ = 1.529; *p* = 0.2223; group × trial interaction effect, *F*_(4,91)_ = 0.47; *p* = 0.7576) and post hoc analysis revealed an immediate update in dopamine release when comparing the first post-CS+ trial between mock and pellets (*p* = 0.018). Chow dopamine was not, or at least less robustly, different from mock on the first trial but significantly decreased between the first and second trial (*t*_(20)_ = 2.178; *p* = 0.0416). Taken together, value-based CS+ dopamine changes appear to predominantly, but not exclusively, follow model-based processes, as nonspecific (chow) devaluation did not occur fully off-line, instead requiring additional (albeit minimal) reexposure to update.

### Dopamine response to CS+ is rapidly restored by additional CS+/US pairing

**Figure 3.**
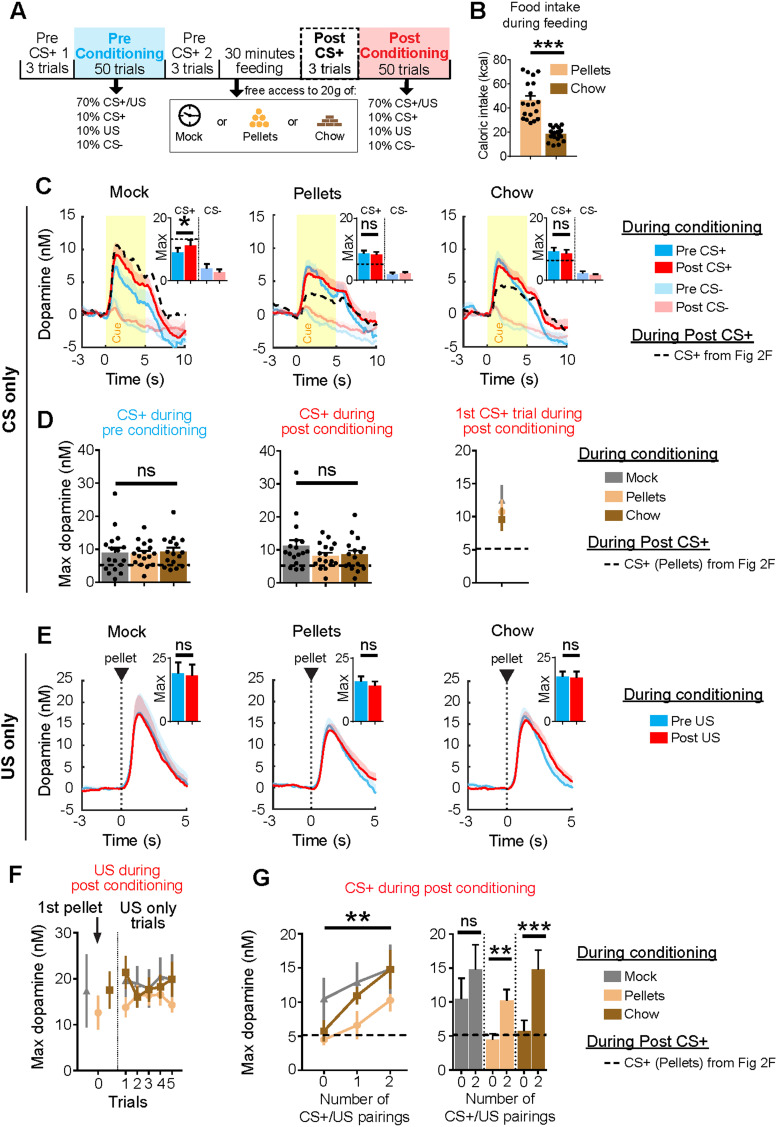
CS+ dopamine is rapidly restored by reintroduction of CS+/US pairings. ***A***, Trial-based schematic where pre- (blue) and post- (red) conditioning phases were evaluated. Post-CS+ trials (dotted line) were used to compare results presented in Figure 2. The conditioning phases consisted of 50 trials, in which 70% were CS+ and US paired trials (35 trials), 10% CS+ (no pellet; 5 trials), 10% US (unpredicted; 5 trials), and 10% CS− (5 trials). Trials were presented in pseudorandomized order on a vITI30 schedule. ***B***, Caloric intake during pellet- or chow-feeding periods. ***C***, Dopamine release across CS trials (5 trials) during conditioning in mock (left), pellet (middle), and chow (right) feeding conditions. Dopamine peak concentrations are shown as insets. Yellow box denotes CS duration. ***D***, Dopamine concentration in CS+ trials during preconditioning (left) and postconditioning (middle). By the first CS+-only trial (right), dopamine has already increased relative to post-CS+ pellet (Fig. 2*F*, middle, dotted line), indicating that CS+ response rapidly restores after pellet and chow-feeding treatment. ***E***, Dopamine concentration to unpredicted US presentation (5 trials) during conditioning in mock (left), pellet (middle), and chow (right) feeding conditions. Dopamine is shown in insets. ***F***, Dopamine in response to the first pellet exposure (either CS+/US pairing or US-only trial) and subsequent US-only trials during postconditioning across feeding conditions. ***G***, Left, Dopamine release across paired CS+/US trials during conditioning after devaluation. Trial 0 is the first CS+ presentation in the conditioning phase (either CS+ only or CS+/US paired trial), whereas the subsequent trials depict dopamine responses to the CS+ during CS+/US paired trials. Right, Comparing Trials 0 and 2 within feeding treatments shows a rapid restoration of the dopamine CS+ response upon reintroduction to the paired CS+/US contingency for both pellet and chow. *p < 0.05; **p < 0.01; ***p < 0.001; ns, not significant.

Next, we investigated how dopamine responds to the reinstated CS+/US contingency, by evaluating CS+ -prompted release during conditioning trials before and after devaluation (Fig. 3*A*). During each conditioning phase, 50 total trials were composed of the following types on a vITI30 schedule: 70% CS+ paired with US, 10% CS+ (no reward), 10% US-only, and 10% CS−. During the feeding period, rats (*n* = 19) consumed large amounts of the offered food, with calorie intake on average being higher for pellets than chow (*W* = −190; *p* < 0.0001; Fig. 3*B*). CS+ presentations elicited a much higher dopamine response than CS− trials on average (Fig. 3*C*), and the dopamine response to CS+ trials during pre- and postdevaluation conditioning differed for the mock treatment (*W* = 101; *p* = 0.0150), but not for pellet (*W* = −19; *p* = 0.6777) or chow (*W* = −24; *p* = 0.5619) treatments. When comparing between groups, there was no difference in dopamine during pre- (*H*_(49)_ = 0.3325; *p* = 0.8468) or postdevaluation (*H*_(49)_ = 1.643; *p* = 0.4399) conditioning phases (Fig. 3*D*, left and middle); in fact, the post-CS+ -diminished dopamine response (Fig. 2*F*) was already restored to predevaluation levels in the first CS+ trial of postconditioning (Fig. 3*D*, right). In Figure 3*E*, (unpredicted) US delivery during the conditioning phase resulted in reliable, high dopamine release uniformly across mock (*W* = −27; *p* = 0.5477), pellet (*W* = −27; *p* = 0.5477), and chow (*W* = −26; *p* = 0.5282) treatments. Furthermore, we compared the dopamine response to the first pellet delivery rats experienced in the postdevaluation conditioning phase (either CS+/US pairing or US-only; *n* = 18 rats), as well as the individual postdevaluation US-only trials, and found dopamine release was stable across time and did not differ between feeding conditions (Fig. 3*F*).

As trial types were pseudorandomized during postdevaluation conditioning, animals had a 70% chance of reexperiencing the CS+/US pairing before their first unrewarded CS+ trial. Since the dopamine response to this first unrewarded CS+ was already reinstated to predevaluation levels (Fig. 3*D*, right), we hypothesized this restoration was driven by the preceding reintroduction to the paired CS+/US contingency. Thus, we quantified the impact of preceding CS+/US pairings on dopamine release in response to subsequent CS+ presentations, by stratifying CS+ dopamine responses according to the number of CS+/US pairings that preceded them, limiting this analysis (Fig. 3*G*, left) to rats (*n* = 15) who were presented a CS+ in their first conditioning trial after devaluation (CS+/US or CS+ trial types; number of preceding CS+/US pairings = 0). We found dopamine release in response to the CS+ during subsequent CS+/US presentations indeed restored rapidly, within two CS+/US paired trials, for both devaluation types [Fig. 3*G*, right; experimental pellets (0 vs 2, *t*_(11)_ = 4.056, *p* = 0.0019); homecage chow (0 vs 2, *t*_(10)_ = 4.638, *p* = 0.0009; mock, *t*_(8)_ = 1.969, *p* = 0.0844)] and differed between devaluation types overall (Fig. 3*G*, left; main effect of group, *F*_(2,84) _= 4.877; *p* = 0.0099; main effect of trial, *F*_(2,84)_ = 0.0028; group × trial interaction, *F*_(4,84)_ = 0.384; *p* = 0.8195). Thus, while CS+ dopamine diminishes fast after satiety (Fig. 2), it reinstates rapidly, while reward dopamine is resistant to change.

## Discussion

In this study, we investigate the dynamics of value-driven changes in dopamine signals, by evaluating how both sensory-specific and nonspecific reward devaluation affects reward/prediction elicited dopamine release in the nucleus accumbens core. First, we demonstrate that our reward–devaluation procedure was effective, as the animals’ motivation to work for food diminished substantially after unlimited access to food for 30 min. This effect was more pronounced after unlimited access to the experimental pellets than to homecage chow. Second, we demonstrate that our pavlovian-conditioning procedure was effective (and completed by the time dopamine recordings were performed), as behavioral response to the CS+ plateaued and behavioral response to the CS− was attenuated. Furthermore, dopamine-signal magnitude induced by the CS+ did not differ before and after a pavlovian-conditioning session. Together, this illustrates that the number of CS–US pairings was sufficient to induce full-task acquisition. Overall, we find that dopamine signals in response to the food-predictive CS+ were diminished instantly by both sensory-specific and nonspecific devaluation, whereas dopamine signals in response to the US remained unaffected by devaluation. Furthermore, CS+ dopamine signals reinstated promptly after additional CS–US pairing.

### Rapid devaluation effects on CS+ dopamine

Activity of dopamine-neuron cell bodies (Schultz et al., 1997) and axons in the striatum (Tsutsui-Kimura et al., 2020), as well as dopamine release from these axons (Day et al., 2007; Hart et al., 2015), can resemble RPE signals. Whether these dopamine RPE signals adhere to model-free or model-based principles is under active debate (Gardner et al., 2018). In a previous study (van Elzelingen et al., 2022a), we investigated the effects of satiety on food delivery-induced striatal dopamine release by comparing dopamine between food-restricted and sated states (induced by the same sensory-specific devaluation as used here). We reported that satiety state was reflected in medial regions of the striatum, including the nucleus accumbens core (as targeted here), where sensory-specific satiety reduced food delivery-induced dopamine release immediately. Thus, this process did not seem to embody a canonical model-free RPE, because model-free processes are assumed to not be instantaneous and require re-experience of the devalued reward (Gardner et al., 2018). Instead, these dopamine changes were consistent with a model-based process, which assumes instantaneous effects without a re-experience requirement (Gardner et al., 2018; Sharpe et al., 2020). However, in our previous experiment, we did not control for effects of general satiety, nor did we condition a previously neutral stimulus that predicted reward delivery. Both points were addressed in the present study by using nonspecific devaluation (homecage chow) and by using an auditory CS, respectively. In the present study, devaluation reduced CS-induced dopamine release instantly without animals reexperiencing pairings of CS with the devalued reward US, consistent with our previous work (van Elzelingen et al., 2022a). This rapid decrease in CS dopamine further supports the idea that value-driven changes in dopamine dynamics are influenced by a model-based mechanism.

It is well-established that the dopamine system is influenced by both physiological state and nutritional content of reinforcers (Ahn and Phillips, 1999; Bassareo and Di Chiara, 1999; Beeler et al., 2012; Ferreira et al., 2012; McCutcheon et al., 2012). Reward devaluation is reported to affect dopamine signaling, with studies demonstrating satiety-attenuated activity in the mesostriatal dopamine system, including reduced dopamine-neuron burst firing (Branch et al., 2013), food-evoked phasic dopamine release (Cone et al., 2014), and dopamine efflux measured with microdialysis (Ahn and Phillips, 1999; Ostlund et al., 2011). Other studies demonstrate that reward devaluation decreases CS-induced dopamine, as reported here (Aitken et al., 2016; Papageorgiou et al., 2016; Gómez-A et al., 2022). However, the study by Aitken and colleagues differs from ours substantially in experimental design and focus, as it evaluated dopamine dynamics during pavlovian-to-instrumental transfer and does not report dopamine release on a single-trial basis, without which instantaneous devaluation effects cannot be identified (Aitken et al., 2016). The study by Papageorgiou and colleagues is more similar to our study but used an experimental design with instrumental access to two types of reward that were devalued separately, without a mock treatment condition. Papageorgiou and colleagues report that an aspect of CS-evoked dopamine was satiety-attenuated instantly (without the requirement to re-experience the CS in the sated state); however, this aspect reflected both CS presentation and behavioral response to the operant lever (presumably shaped by incentive learning in the devalued state). Therefore, although ample evidence illustrates that the dopamine system is affected by satiety, our findings are the first to demonstrate that the influence of reward devaluation on reward-associated CS is instant (and, thus, presumably proceeds via a model-based mechanism).

### Dopamine is decreased irrespective of devaluation-stimulus identity

An important qualifier of our finding that reward devaluation instantly reduces CS-induced dopamine release (without animals reexperiencing pairings of CS with devalued reward) is that we observed these dynamics irrespective of whether the devaluation procedure was sensory-specific or nonspecific. We expected to see a larger effect of pellet devaluation on CS-induced dopamine compared with chow, as the former affects both pellet-specific appetite and hunger, whereas the latter affects hunger only. Because both food pellets and chow have a similar effect, the process underlying changes in dopamine cannot be fully model-based, as model-based processes are assumed to encode stimulus identity. Our finding is consistent with that of Papageorgiou and colleagues, who report a similar reward-unspecific reduction in CS-induced dopamine release following reward devaluation (Papageorgiou et al., 2016). Additionally, the effects of our devaluation on dopamine release highlight that our behavioral and neurochemical results are not fully aligned or that their dynamics are at least nonlinear (Hollon et al., 2014): despite a lower calorie consumption of chow (during the 30 min feeding procedure), both food types had similar overall effects on dopamine.

An open question in this context is whether the chow is more filling than pellets and more effortful to chew and digest because of its shape and consistency or whether the fact that it is identical to what the rats are fed in the home cage may make it less attractive. We found that chow devaluation only differs from pellet devaluation in that its trial-by-trial temporal dynamics of dopamine release are slower: Figure 2*H* (right) and Figure 3*G* suggest, respectively, that (compared with pellet-), chow-based devaluation requires a brief period of reexposure to the devalued reward and produces a less robust/long-lasting decrease in dopamine. However, it is possible that both the differential intake and effect on lever pressing after chow devaluation may have contributed to these differences in the first-trial CS+ dopamine responses. Taken together, although the immediate effect of prefeeding on CS dopamine violates model-free assumptions (i.e., values/“weights” were adjusted in an experience-independent manner), the lack of identity-selective dopamine effects violates model-based assumptions. Thus, activity of the mesolimbic dopamine system cannot be described as either model-based or model-free exclusively but instead may operate according to a mix of both theoretical constructs.

### US dopamine is persistent and may reinstate CS dopamine

Another finding that undermines the dichotomous model classification of mesolimbic dopamine is that, in contrast to CS-dopamine reduction, our devaluation did not decrease food pellet-induced dopamine release. This finding appears inconsistent with our previous work (van Elzelingen et al., 2022a) where we reported an immediate decrease in reward-induced dopamine when comparing hungry and prefed animals. There are several potential explanations for this discrepancy, predominantly due to dissimilarities in the behavioral paradigms, including the timing of devaluation with regard to recording, the type of devaluation control (mock vs no-prefeeding), the order in which rats were presented CS and US after devaluation, the duration of training, and time spent waiting in the operant box before behavioral sessions were started. However, we believe the most consequential difference is the timing of this US-evoked dopamine response in a broader sense: in our previous work, devaluation occurred before the start of the session, and its effects on US dopamine were evaluated as response to unpredicted pellets administered at the start of the session (whereas in the present study, devaluation occurred in the middle of the session and unpredicted pellets were delivered in the final block of the session). In other words, US dopamine in our previous work not only reflects the value of a food pellet but presumably also the value of the entire session. Speculatively, this indicates that presession pellets are (at least partially) governed by a model-based process, whereas within-session pellets are (exclusively) model-free.

In line with this idea, although they did not measure reward delivery-induced dopamine alone, Papageorgiou and colleagues report that expected reward delivery-induced dopamine (i.e., US preceded by a CS) was not decreased by reward devaluation (Papageorgiou et al., 2016). They also report that devaluation-diminished CS dopamine returned to predevaluation levels swiftly, which is consistent with our finding that CS dopamine reinstates within a few CS+/US pairings. We presume that the devaluation-resistant, persisting US dopamine signal crucially enhances the speed at which CS-induced dopamine is reinstated by postdevaluation reconditioning. This provides an intriguing illustration of a model-free process associated with reward delivery steering a model-based process for CS dopamine.

In summary, we find dopamine-signal magnitude responds to decreased reward value through several distinct adaptations: CS dopamine diminishes but reinstates fast, whereas reward dopamine is resistant to change and may contribute to swift reinstatement of the former; specific and nonspecific devaluation yielded similar results; thus these adaptations are not selective for stimulus identity. As a whole, this matches a framework where the dopamine system is governed, in the context of changing reward values, by a mix of model-based and model-free mechanisms. Alternatively, the two mechanisms may be in a state of fluent exchange or may be separated temporally or in an occasion-bound manner.

## Synthesis

Reviewing Editor: Jibran Khokhar, Western University Department of Anatomy and Cell Biology Decisions are customarily a result of the Reviewing Editor and the peer reviewers coming together and discussing their recommendations until a consensus is reached. When revisions are invited, a fact-based synthesis statement explaining their decision and outlining what is needed to prepare a revision will be listed below. The following reviewer(s) agreed to reveal their identity: NONE.

This is a well designed paper that provides compelling evidence that reward devaluation rapidly reduces dopamine release in response to reward-predictive cues, but not to the reward itself, regardless of the devaluation being sensory-specific or non-specific. Additionally, reconditioning with the devalued reward quickly restores dopamine responses to the cues back to pre-devaluation levels. The authors have done an excellent job of addressing all the reviewer comments, making the manuscript acceptable as is.
